# Assessment of TSPO Gene Expression Levels in Colorectal Cancer Tumors: A Paired Sample Study

**DOI:** 10.1002/cnr2.70256

**Published:** 2025-06-23

**Authors:** Hamidreza Karami Gorji, Mehdi Karimi, Masoud Mortezazadeh, Niyousha Shirsalimi, Sheyda Akhshabi, Alireza Yousefi Ladmakhi, Mehdi Kashani, Seyyed Taher Seyyed Mahmoudi, Abbas Mofidi, Abolhasan Rezaei

**Affiliations:** ^1^ Department of Genetics Islamic Azad University, Tonekabon Branch Tonekabon Iran; ^2^ Faculty of Medicine Bogomolets National Medical University (NMU) Kyiv Ukraine; ^3^ Department of Hematology‐Oncology, Cancer Institute, Imam Khomeini Hospital Complex Tehran University of Medical Sciences (TUMS) Tehran Iran; ^4^ Faculty of Medicine Hamadan University of Medical Science (UMSHA) Hamadan Iran; ^5^ Department of Internal Medicine, Sina Hospital Tehran University of Medical Sciences (TUMS) Tehran Iran; ^6^ Faculty of Medicine Tabriz University of Medical Sciences Tabriz Iran; ^7^ School of Medicine Iran University of Medical Sciences (IUMS) Tehran Iran

**Keywords:** colorectal cancer, genetics, medical genetics, molecular biology, oncology, TSPO gene

## Abstract

**Background and Aim:**

Colorectal cancer (CRC) is the second leading cause of cancer‐related deaths. Early detection through screening is crucial for improving treatment outcomes. Advanced stages of CRC are frequently associated with distant metastasis, posing significant challenges to treatment. This study aimed to assess Translocator Protein (TSPO) gene expression as a potential indicator of invasive behavior in CRC patients. It explored its utility as a biomarker for CRC diagnosis and prognosis.

**Methods:**

In this case–control study, 50 samples were collected from 25 patients with CRC who had undergone colectomy. These included 25 colorectal tumor tissues (case) and 25 non‐tumor marginal tissues (control). RNA was extracted and assessed for TSPO gene expression using Real‐Time Polymerase Chain Reaction (RT‐PCR). The data were analyzed using Relative Expression Software Tool (REST) to determine gene expression levels. SPSS version 24 was used for all statistical analyses.

**Results:**

A significant increase in TSPO gene expression was observed in CRC tumor tissues compared to normal samples (*p* < 0.001). This elevated expression was significantly associated with tumor grade (*p* < 0.05), suggesting a link with disease severity. However, no significant differences in TSPO expression were found between tumor and non‐tumor groups when analyzed by sex (male vs. female) or age (< 50 vs. > 50 years).

**Conclusion:**

TSPO gene expression is elevated in colorectal tumor tissues and is significantly associated with tumor grade. These findings suggest a potential role for TSPO in the development and progression of colorectal cancer. Recognizing the relationship between these genes in the differentiation of changes in CRC cells, especially in clinical trials, can be crucial in finding and controlling the mechanisms involved.

**Abbreviations**
CRC: Colorectal Cancer
CT: Cycle threshold
GAPDH: Glyceraldehyde‐3‐phosphate dehydrogenase
MT: Melting temperature
PCR: Polymerase Chain Reaction
qRT‐PCR: Quantitative Reverse Transcription Polymerase Chain Reaction
RT‐PCR: Real‐Time Polymerase Chain Reaction
TSPO: Translocator Protein

## 1 Introduction

Colorectal cancer (CRC) is one of the most prevalent and severe malignancies worldwide, accounting for 10% of total cancer incidence and 9.4% of all cancer‐related fatalities. In 2020, 1.93 million new cases were identified, and nearly 1 million deaths occurred. Projections suggest that new cases will reach 3.2 million by 2040 [1, 2]. Despite the positive outlook for CRC therapy, the increasing number of cases and younger generations pose a significant public health challenge [3, 4, 5].

Despite advances in screening and treatment, the prognosis for advanced CRC remains poor, primarily due to its aggressive nature and propensity for metastasis [6]. Hence, there is an urgent need for alternative CRC screening methods that are cost‐effective, noninvasive, easily measurable, and accurate. Identifying biomarkers in CRC has become crucial for early disease detection, prognostic stratification, surveillance, and therapy selection [7, 8, 9]. Identifying molecular markers that can predict disease behavior, guide treatment decisions, and improve patient outcomes is a critical area of ongoing research [10].

Advancements in genomics, transcriptomics, proteomics, and metabolomics have contributed to the identification of a larger number of potential biomarkers and enhanced our understanding of the development of CRC and its molecular signatures [11, 12, 13]. One such molecular marker of interest is the Translocator Protein (TSPO), previously known as the peripheral benzodiazepine receptor [14, 15]. TSPO is a protein located on the outer mitochondrial membrane and is involved in various cellular functions, including cholesterol transport, steroid biosynthesis, apoptosis, and cellular proliferation. Overexpression of TSPO has been observed in numerous malignancies, suggesting a potential role in cancer pathogenesis and progression [15, 16, 17].

TSPO has been explored as a target for positron emission tomography (PET) imaging in CRC. Radiolabeled TSPO ligands, such as [18F] FEPPA, have shown promise in non‐invasively quantifying TSPO levels in tumors. This imaging approach could be used for accurate diagnosis, disease progression monitoring, and therapy response assessment in CRC patients [18, 19].

The precise role of TSPO in CRC remains underexplored, yet emerging evidence suggests that its expression may correlate with tumor aggressiveness and metastatic potential [14]. Several studies have investigated the expression of the TSPO protein levels in colorectal malignancies [20, 21, 22], meningiomas [23], glioma [24], glioblastoma [25], hepatocellular carcinoma [26], melanoma [27], and breast cancer [28], indicating that TSPO could play a role these cancers. Furthermore, the expression of this protein has shown a positive correlation with the progression of certain cancers while exhibiting a negative association with the prognosis of certain other cancers [20, 21, 22, 23, 24, 25, 26, 27]. However, the mechanisms by which TSPO contributes to CRC invasiveness and its potential utility in clinical practice require further investigation.

The aim of designing and conducting this study is to investigate the expression level of the TSPO gene in patients with CRC. Through a comprehensive assessment of TSPO levels in CRC patients, we aim to elucidate its potential role in cancer progression and evaluate its viability as a diagnostic and prognostic tool.

## 2 Materials and Methods

### 2.1 Study Design, Setting, Ethics

This case–control study was conducted at the *Oncology Research Center of Imam Khomeini Hospital* in Tehran, Iran, from February to June 2018. The study protocol was approved by the hospital's Ethics Committee and followed its guidelines. Written informed consent was obtained from all participants using the hospital‐approved consent form. Participants' privacy and confidentiality were strictly protected throughout the study, and all procedures were carried out in accordance with ethical standards.

A total of 25 patients with CRC were included in this study. None of the patients had undergone chemotherapy before sample collection. All patients were examined by an expert team of physicians, including an internist, oncologist, and pathologist, and diagnostic and pathological tests confirmed colorectal cancer. After confirmation of the CLC, the patients were candidates for colectomy.

### 2.2 Tissue Sampling and Preparation

Two tissue samples were obtained from each patient who had undergone colorectal surgery (colectomy), including tumor tissue samples and adjacent non‐tumor (ANT) tissue samples from the same patients. A total of 50 tissue samples were classified into two groups: tumor tissue samples (Case) and non‐tumor tissue samples (Control).

During sample collection, colorectal tissue from cancerous and noncancerous colorectal tissue was collected in paraffin‐embedded tubes and stored at −80°C. Before total RNA extraction, the tissue was physically minced, and lysis buffer was added. The collection and preparation of samples from both non‐tumor control and tumor tissues were performed in full compliance with health principles and to the participants' satisfaction.

### 2.3 RNA Extraction and Qualitative/Quantitative Analysis

The YEKTA Equipment kit was utilized for RNA extraction based on the tissue sample protocol. The entire process was conducted in an isolated environment under a hood to prevent contamination. To ensure all materials and equipment were free of RNase, containers and tubes used for working with RNA were treated with 1% DEPC solution for 1 h at 37°C or overnight at room temperature. Subsequently, they were sterilized by autoclaving at 15 psi for 15 min.

The quantity and purity of the extracted RNA were determined using the optical density (OD) method with a NanoDrop spectrophotometer (Thermo Scientific—NanoDrop 2000).

### 2.4 Determination of the Quality and Quantity of Extracted RNA

**FIGURE 1:**
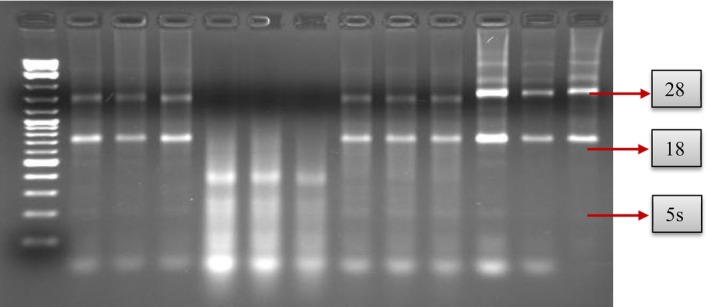
Agarose gel electrophoresis of RNA samples on a 1.5% agarose gel. The 100 bp molecular ladder (leftmost lane) serves as a size reference. The distinct 28S, 18S, and 5S ribosomal RNA bands indicate RNA integrity. Clear and well‐defined bands confirm the successful extraction and quality of the RNA samples.

After RNA extraction, its quality was assessed by electrophoresis on a 1.5% agarose gel to ensure integrity. The presence of distinct 18S, 28S, and 5S ribosomal bands, as shown in Figure 1, confirmed successful extraction. RNA samples were mixed with a loading buffer, loaded into gel wells, and subjected to electrophoresis. The gel was then visualized under ultraviolet light using the Gel. Doc Analyze Transilluminator to verify RNA integrity.

### 2.5 Primer Design and Preparation

Primer sequences were first analyzed using OligoAnalyzer software to perform the PCR and subsequently validated with the NCBI BLASTn program to select the most appropriate sequences. To prevent the amplification of genomic DNA during RT‐PCR, primers were designed to locate the upstream and downstream primers on separate exons. This design allows for the distinction of PCR products contaminated with genomic DNA based on the differing sizes of the amplified fragments, thereby verifying gene expression at the mRNA level. The primers used in this assay for the TSPO gene and glyceraldehyde‐3‐phosphate dehydrogenase (GAPDH) were synthesized and stored at −20°C (Table 1).

**TABLE 1:** The sequence of primers used for the amplification of the Translocator Protein (TSPO) and glyceraldehyde‐3‐phosphate dehydrogenase (GAPDH) genes.

Genes	Primer type	Primer sequence
TSPO	Forward primer	5′‐TGTGAAACCTCCCAGCTCTTGGCA‐3′
	Reverse primer	5′‐TAGCTTGCAGAAACCCTCTTTCCA‐3′
GAPDH	Forward primer	5′–ATCACTGCCACTCAGAAGAC–3′
	Reverse primer	5′–ACATTGGGGGTAGGAACAC–3′

### 2.6 cDNA Synthesis and Validation

The Takara kit was utilized for cDNA synthesis. All extracted RNAs were initially adjusted to a concentration of 1000 ng/μL based on NanoDrop measurements. The nucleotide strands were denatured and then cooled, followed by adding 10 μL of the RT Master Mix enzyme. The reaction mixture was processed in a PCR machine according to the kit's temperature program: 25°C for 10 min (primer annealing), 42°C for 60 min (cDNA synthesis), and 70°C for 10 min (enzyme inactivation).

To verify the quality of the synthesized cDNA, RT‐PCR was performed using the GAPDH gene as an internal control. The PCR products were analyzed by electrophoresis on a 2% agarose gel at 100 V, revealing a distinct 219 bp band corresponding to the GAPDH primer, confirming accurate cDNA synthesis. A negative control without reverse transcriptase was included in both RT‐PCR and qRT‐PCR to rule out genomic DNA contamination, while a no‐template control was used to assess PCR material contamination. Additionally, primers were designed to span different exons, and a housekeeping gene served as a positive control to validate cDNA integrity.

### 2.7 Investigation of Gene Expression Using RT‐PCR

The Polymerase Chain Reaction (PCR) method with SYBR Green (from Takara Company) was used to investigate changes in gene expression. The procedure involved several key steps. Initially, a master mix containing 9 μL of SYBR Green, 8 μL of nuclease‐free water, 1 μL of forward primer, and 1 μL of reverse primer was prepared for the required number of reactions. An additional sample served as a negative control to rule out contamination and minimize potential pipetting errors. Due to the complexity of the experiment and the extensive number of samples, a meticulous approach was adopted following gentle centrifugation to ensure thorough mixing. Each set of reactions for specific samples and genes was allocated into individual tubes within eight‐tube strips, with each tube receiving 19 μL of reaction mix. Subsequently, 1 μL of cDNA was added to each tube, bringing the final reaction volume to 20 μL, with each sample run duplicated (Data S1).

Two methods were employed to validate the accuracy and specificity of the RT‐PCR reaction for the target gene. Firstly, a melt curve analysis was performed post‐reaction, plotting melting curves of products from 55°C to 95°C to identify specific melting peaks indicative of the target gene. The melting temperature of the amplified gene fragments was determined to be 60°C, and this temperature was thus used as the annealing temperature (Table 2). Secondly, to confirm the specificity of the RT‐PCR products, the final PCR products were loaded onto a 2% agarose gel and subjected to electrophoresis. This step ensured the amplification of specific gene regions without the presence of nonspecific products or primer dimers.

**TABLE 2:** Time and temperatures used in real‐time polymerase chain reaction.

No.	Steps	Temperature	Time	No. of cycles
I	Initial denaturation	95°C	30 S	1
II	Denaturation	95°C	10 S	38
III	Annealing	60°C	32 S	38
VI	Elongation	55°C–95°C	34 S	38

### 2.8 Validation of RT‐PCR

Using synthesized cDNAs, RT‐PCR reactions were performed for all samples using the reference gene GAPDH. After completing the reactions, the samples' cycle threshold (CT) values were extracted from the RT‐PCR device analysis. The results obtained from these samples indicated that the products of the examined genes were specific, each displaying its distinct melting temperature, thereby confirming the accuracy of the primers and the validity of RT‐PCR execution.

### 2.9 Quantitative Analysis of the RNAs

The quantitative evaluation of the RNAs was performed using the results obtained from the *NanoDrop device*. An appropriate OD range (400–600 ng/μL) and a ratio > 1.8–2 indicated that the RNAs were high‐quality and free from protein contamination (Table 3).

**TABLE 3:** Quantitative analysis of RNA using the NanoDrop device.

Sample name	OD	260/230	280/260
A01336101	421	2.35	1.87
A01232101	860	2.34	2.1
A01281101	560	2.23	1.94
A01300101	647	2.19	2.06
A01355101	940	2.45	2.11

### 2.10 Qualitative Analysis of Gene Expression

Amplification curves were successfully generated for all samples using TSPO primers. The curves exhibited typical exponential growth patterns, indicating successful amplification of the TSPO gene target. The CT values obtained from these curves can be used to quantify the initial amount of TSPO mRNA in each sample.

### 2.11 ΔCT Calculation Method

Gene expression levels were analyzed using the ΔCT method. To calculate ΔCT, the CT values for TSPO and GAPDH were subtracted (ΔCT = CT(TSPO) − CT(GAPDH)), with lower ΔCT values indicating higher expression. This calculation was performed for both healthy and tumor samples. The ΔΔCT was then calculated as the difference between the ΔCT values of the tumor and healthy samples (ΔΔCT = ΔCT(tumor) − ΔCT(healthy)), with lower ΔΔCT indicating higher expression. Fold change was determined using the formula Fold change = 2^(−ΔΔCT)^, where a higher fold change reflects increased expression.

### 2.12 Statistical Analysis

The gene expression data obtained from RT‐PCR were analyzed using the Relative Expression Software Tool software to assess the TSPO gene expression levels in the tumor and tumor margin. The normality of the data distribution was evaluated with the one‐sample Kolmogorov–Smirnov test. Subsequently, one‐way ANOVA was employed to examine the differential expression levels of the TSPO gene across various tumor sizes. A significance threshold was set at *p*‐value < 0.05. All statistical analyses were conducted using SPSS version 24. RT‐PCR and CT values were analyzed to assess TSPO gene expression. The ΔCT values for tumor and non‐tumor samples were tested for normality using the One‐sample Kolmogorov–Smirnov test (*p* > 0.05).

## 3 Result

### 3.1 Patient Characteristics (Descriptive Statistics)

This study analyzed 50 samples collected from 25 CRC patients who underwent colectomy. The samples comprised 25 (50%) tumor tissue samples and 25 (50%) adjacent healthy tissue samples. The participants' ages ranged from 28 to 73, with a mean age of 55 ± 22. The majority of patients were males (64%, *n* = 32), and the rest were females (36%, *n* = 18). This balanced distribution of cases and controls, along with the diverse age range and gender representation, provided a comprehensive basis for evaluating differences in TSPO gene expression levels.

### 3.2 TSPO Gene Expression Level

Statistical analysis revealed a highly significant difference in TSPO gene expression in CRC tumor samples compared to normal tissue samples (*p* < 0.001). In addition, TSPO gene expression levels were analyzed in both CRC tumor and non‐tumor tissues based on gender, age, tumor grade, and cancer stage. The mean TSPO gene expression levels and corresponding *p*‐values are summarized in Table 4.

**TABLE 4:** The mean of Translocator Protein (TSPO) gene expression in colorectal cancer tumor and non‐tumor tissue samples.

Mean TSPO gene expression			
Adjusted	Variables	Tumor (*n* = 25)	Non‐tumor (*n* = 25)
Gender	Male	9.39	7.58
	Female	11.10	7.39
	*p*‐value	0.689	0.948
Age (years)	< 50	9.00	8.84
	≥ 50	10.35	7.34
	*p*‐value	0.688	0.832
Grades	Grade I	6.99	5.55
	Grade II	9.30	6.45
	Grade III	16.86	10.89
	Grade IV	18.39	13.40
	*p*‐value	< 0.001*	0.123
Stages	Stage 0	19.42	16.26
	Stage I	1.60	7.33
	Stage II	7.86	4.11
	Stage III‐a	12.53	9.72
	Stage III‐b	10.05	0.82
	Stage IV	9.22	7.31
	*p*‐value	< 0.001*	0.010

*Note:* The Bold values represent the statistical significance. *p*‐value < 0.05 = significance.

### 3.3 TSPO Gene Expression Based on Tumor Grades

**FIGURE 2:**
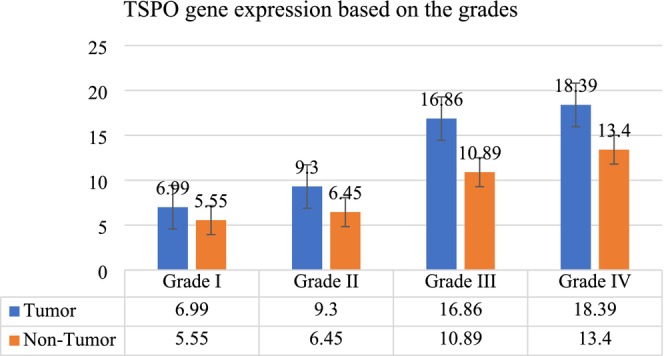
The mean of Translocator Protein (TSPO) gene expression level in tumor and non‐tumor tissue samples adjusted by grade.

Grade I tumors showed a mean TSPO expression of 6.99 in tumor tissues compared to 5.55 in non‐tumor tissues. Grade II tumors had mean expressions of 9.30 in tumor tissues and 6.45 in non‐tumor tissues. Grade III tumors displayed significantly higher TSPO expression, with a mean of 16.86 in tumor tissues and 10.89 in non‐tumor tissues. Grade IV tumors also had elevated TSPO expression, with a mean of 18.39 in tumor tissues and 13.40 in non‐tumor tissues. The *p*‐values indicated a significant increase in TSPO expression with higher tumor grades (*p <* 0.001 for tumor tissues and *p* = 0.123 for non‐tumor tissues) (Figure 2).

### 3.4 TSPO Gene Expression Based on Cancer Stages

**FIGURE 3:**
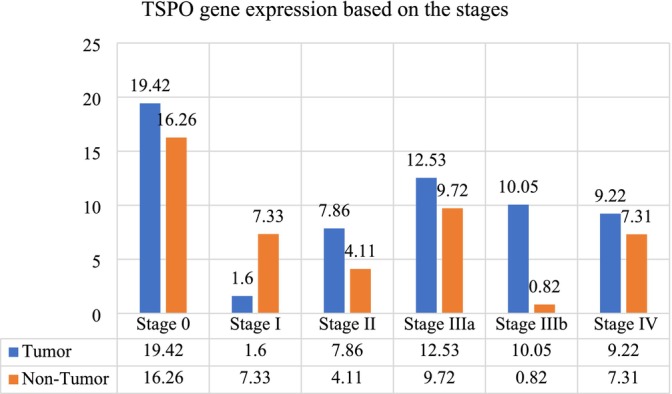
The mean of Translocator Protein (TSPO) gene expression level in tumor and non‐tumor tissue samples adjusted by stage.

Stage 0 tumors had mean TSPO expressions of 19.42 in tumor tissues and 16.26 in non‐tumor tissues. Stage I tumors showed a marked decrease, with mean expressions of 1.60 in tumor tissues and 7.33 in non‐tumor tissues. Stage II tumors exhibited mean expressions of 7.86 in tumor tissues and 4.11 in non‐tumor tissues. Stage IIIa tumors had mean expressions of 12.53 in tumor tissues and 9.72 in non‐tumor tissues. Stage IIIb tumors had mean expressions of 10.05 in tumor tissues and 0.82 in non‐tumor tissues. Stage IV tumors had mean expressions of 9.22 in tumor tissues and 7.31 in non‐tumor tissues. The *p*‐values for these comparisons were significant (*p* = 0.001 for tumor tissues and *p* = 0.010 for non‐tumor tissues), indicating significant variations in TSPO expression across different cancer stages (Figure 3).

### 3.5 TSPO Gene Expression Based on Age and Gender

Patients under 50 years exhibited mean TSPO expression levels of 9.00 in tumor tissues and 8.84 in non‐tumor tissues. Patients aged 50 and above had mean TSPO expression levels of 10.35 in tumor tissues and 7.34 in non‐tumor tissues. The *p*‐values for these comparisons were 0.688 and 0.832, respectively, showing no significant differences in TSPO expression with age (Figure S1–S2, Table 4).

The mean TSPO gene expression in male patients was 9.39 in tumor tissues and 7.58 in non‐tumor tissues. The mean TSPO expression in female patients was 11.10 in tumor tissues and 7.39 in non‐tumor tissues. The *p*‐values for male and female groups were both greater than 0.05 (0.689 and 0.948, respectively), indicating no statistically significant differences in TSPO expression based on gender (Figure S3, Table 4).

## 4 Discussion

The current study aimed to assess the levels of the TSPO gene expression in both tumor and non‐tumor tissue samples obtained from patients with CRC, considering variables including gender, age, tumor grades, and cancer stages. For this purpose, the data were obtained from analyzing 25 tumor tissue samples and 25 ANT tissue samples from the same patients with CRC. The study findings revealed a highly significant difference in TSPO gene expression between CRC tumor samples and normal tissue samples. In addition, TSPO gene expression was significantly higher in tumor tissues compared to non‐tumor tissues, with expression levels increasing with tumor grade and varying significantly across cancer stages. However, no significant differences in TSPO expression were observed based on age or gender. The findings provide significant insights into the potential role of TSPO as a biomarker for tumor progression in colorectal cancer.

Notably, in this study, we found that TSPO gene expression levels increased with tumor grade in both CRC tumors and adjacent healthy tissue samples, whereas they decreased with advancing cancer stage. This pattern suggests a complex role of TSPO in CRC progression. At the molecular level, TSPO is involved in mitochondrial function, reactive oxygen species regulation, and apoptosis [28]. In high‐grade tumors, which are poorly differentiated and more aggressive, elevated TSPO expression may reflect increased mitochondrial activity and metabolic reprogramming to support rapid proliferation [29, 30]. Additionally, TSPO is linked to inflammation and lipid metabolism [30], both of which are heightened in aggressive tumors. However, as CRC advances in stage, tumors often undergo metabolic shifts toward a more hypoxic, immune‐evasive, and invasive phenotype [13, 31]. The downregulation of TSPO in later stages may be associated with cancer cell adaptation to low‐oxygen environments, decreased apoptosis susceptibility, and a transition to mesenchymal‐like states that favor invasion and metastasis [32]. Furthermore, TSPO‐related mitochondrial dysfunction in advanced stages may contribute to a metabolic shift toward glycolysis (Warburg effect), leading to reduced TSPO expression [33]. Thus, TSPO expression in CRC appears to be stage‐dependent, with initial upregulation facilitating tumor growth while later downregulation supports survival and metastatic potential.

The observed correlation between TSPO expression and tumor grade aligns with previous similar studies. For instance, Liu et al. [20] demonstrated higher TSPO expression in CRC tissues and cells compared to ANT samples and normal intestinal epithelial cells. Overexpression of TSPO in CRC cells enhances proliferation and tumor marker expression (CEA and CA19‐9) while inhibiting apoptosis, whereas TSPO interference results in reduced proliferation and increased apoptosis [20].

Similarly, our findings are supported by McKinley et al. [34], who found that 64% of colon cancers showed positive TSPO expression, with higher TSPO levels significantly associated with higher tumor grades. Their study demonstrated that patients with elevated TSPO expression were three times more likely to have grade 3 tumors compared to grade 2 tumors [34]. This reinforces our observation of increased TSPO expression correlating with tumor aggressiveness. Interestingly, Königsrainer et al. [21] reported differential TSPO expression between colon and rectum carcinomas, suggesting distinct biological behaviors of these tumor types. This highlights the need for further studies to explore the tissue‐specific roles of TSPO in CRC.

Furthermore, TSPO's involvement in metabolic reprogramming, as demonstrated by Wu et al. [35], suggests a mechanism by which TSPO may facilitate liver metastasis of CRC. Wu et al. [35] showed that TSPO‐induced metabolic reprogramming drives liver metastasis by activating lysine degradation and regulating chromatin modifiers, thereby enhancing the metastatic potential of CRC cells.

Shoukrun et al.'s findings on the role of TSPO in apoptosis provide a potential therapeutic angle. They showed that TSPO knockdown increased malignancy in vitro, whereas its agonist FGIN‐1‐27 reduced tumor growth in vivo, suggesting that targeting TSPO could be beneficial in CRC treatment [36]. However, Bhoola et al. highlighted the complexity of TSPO's role across different cancer types. Their study indicated that while TSPO mRNA expression is elevated in liver, prostate, kidney, and brain cancers, it is lower in colon and lung cancers, suggesting tissue‐specific expression patterns and roles [14].

Our findings corroborate the key observations noted in the provided text. Similar to the study's conclusion that TSPO gene expression increases with tumor malignancy, our research also identified elevated TSPO levels in more advanced CRC grades. Additionally, the association between TSPO genetic polymorphism A147T and poorer outcomes in male glioblastoma patients parallels our findings on TSPO's prognostic value. Our analysis also highlights that higher TSPO expression correlates with shorter overall survival and progression‐free survival, particularly in aggressive cancer types [37]. The study by Wongso et al. [17] reinforces the relevance of our results, which reported TSPO upregulation in cancer cells and its association with aggressive phenotypes and poor prognosis. This alignment suggests that TSPO could be an effective target for novel therapeutic strategies and image‐guided surgeries, offering a promising direction for future cancer treatments [17].

The regulation of TSPO expression in CRC involves several signaling pathways and transcription factors, with Protein kinase C epsilon playing a pivotal role through the MAPK pathway. This regulation activates transcription factors such as STAT3 and c‐Jun, which bind to the TSPO promoter and enhance its transcription [38, 39]. TSPO significantly impacts cellular functions crucial for cancer progression; its interaction with voltage‐dependent anion channels influences mitochondrial function and cellular metabolism, which are essential for cancer cell survival and proliferation [40].

Given the role of TSPO in CRC, targeting its expression and function presents a promising therapeutic strategy. Interfering with TSPO expression has been shown to inhibit CRC cell proliferation and promote apoptosis; for instance, remimazolam (Rem) reduces TSPO levels, decreasing cell viability and proliferation while increasing apoptosis in CRC cells [20]. TSPO's elevated expression in CRC and its association with tumor aggressiveness make it a valuable biomarker for diagnostic and prognostic purposes. Imaging techniques such as PET with TSPO‐targeted probes can non‐invasively quantify TSPO levels in tumors, aiding in the assessment of disease progression and treatment response [34].

### 4.1 Clinical Implications

TSPO expression is closely linked to tumor grades and stages in CRC, suggesting its potential as a diagnostic and prognostic biomarker. It could help stratify patients based on tumor aggressiveness, guiding personalized treatment plans. Patients with higher TSPO levels may require more aggressive treatments, while those with lower levels could avoid overtreatment. Additionally, targeting TSPO with inhibitors might slow cancer progression by reducing cell proliferation and promoting apoptosis. However, more research is needed to understand TSPO's role in tumor biology and develop targeted therapies.

### 4.2 Strengths, Limitations, Research Suggestions

This study has several strengths, including its focus on the TSPO gene as a potential CRC biomarker, which could provide valuable insights for early diagnosis and prognosis. RT‐PCR to analyze TSPO gene expression in both tumor and marginal tissue samples is a robust method, ensuring reliable and precise measurement of gene expression levels. However, the study is limited by its relatively small sample size of 25 patients, which may not represent the broader CRC patient population. Additionally, the study only assessed gene expression at the mRNA level and did not extend to protein‐level analysis due to budget constraints. This limits the ability to fully understand the functional implications of increased TSPO expression. Future studies should include larger sample sizes and incorporate protein‐level analysis using methods like immunohistochemistry and Western blotting to validate these findings and explore the clinical utility of TSPO as a biomarker in CRC.

## 5 Conclusion

In conclusion, this study's findings underscore the significance of TSPO in CRC progression. TSPO gene expression levels are markedly higher in malignant tissues compared to ANT tissues. Biomarkers like TSPO play a crucial role in identifying new treatment targets, and their accurate measurement is vital for diagnosing and predicting disease course. However, the discovery of reliable biomarkers remains challenging due to the complex nature of cancer biology. Future research should continue to explore the multifaceted role of TSPO in cancer and its potential as a therapeutic target.

## Author Contributions

H.K.G., A.R., M.M., and M.K. conceived and designed the study. N.S., S.A., and A.Y.L. conducted the experiments and collected data. A.R. and M.M. performed the statistical analysis. M.K. contributed to revising and conceptualizing the manuscript. A.R. and M.M. supervised the project. All authors contributed to the primary drafting and read and approved the final manuscript.

## Ethics Statement

The Ethics Committee of Imam Khomeini Hospital, Tehran, Iran, approved this study. Before their inclusion, all participants provided written informed consent.

## Consent

The authors have nothing to report.

## Conflicts of Interest

The authors declare no conflicts of interest.

## Supporting information


**Data S1.** Supporting information.

## Data Availability

The data that support the findings of this study are available on request from the corresponding author. The data are not publicly available due to privacy or ethical restrictions.
