# Supplementary material for: Assessment of TSPO Gene Expression Levels in Colorectal Cancer Tumors: A Paired Sample Study
Source: Cancer Rep (Hoboken). 2025 Jun 23;8(6):e70256. doi: 10.1002/cnr2.70256 (PMC12185149; doi:10.1002/cnr2.70256)
Supplement: Supplementary file 1 — Data S1. Supporting information. [file CNR2-8-e70256-s001.docx]

**Supplementary files**

**_________________________________________________________________________**

**Supplementary Table 1.** Values used to prepare samples in RT-PCR

| **Component** | **Volume** |
| --- | --- |
| Cyber green | 9 µl |
| Nuclease-free water | 8 µl |
| Forward Primer5 | 1 µl |
| Reverse Primer5 | 1 µl |
| cDNA | 1 µl |
| Final volume | 20 µl |

**Supplementary Figure 1:** RNA extraction quality assessment curve ensuring nano-drop contamination-free samples.


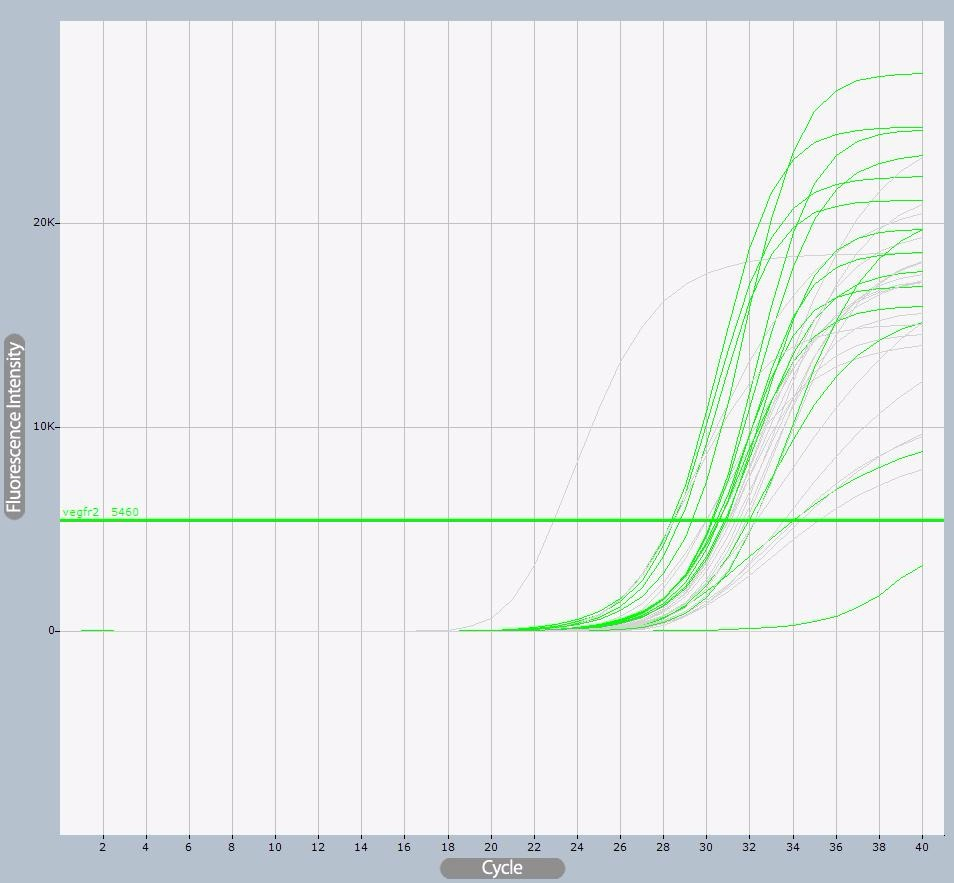


**Supplementary Figure 2.** The amplification threshold curves for samples subjected to TSPO primers.


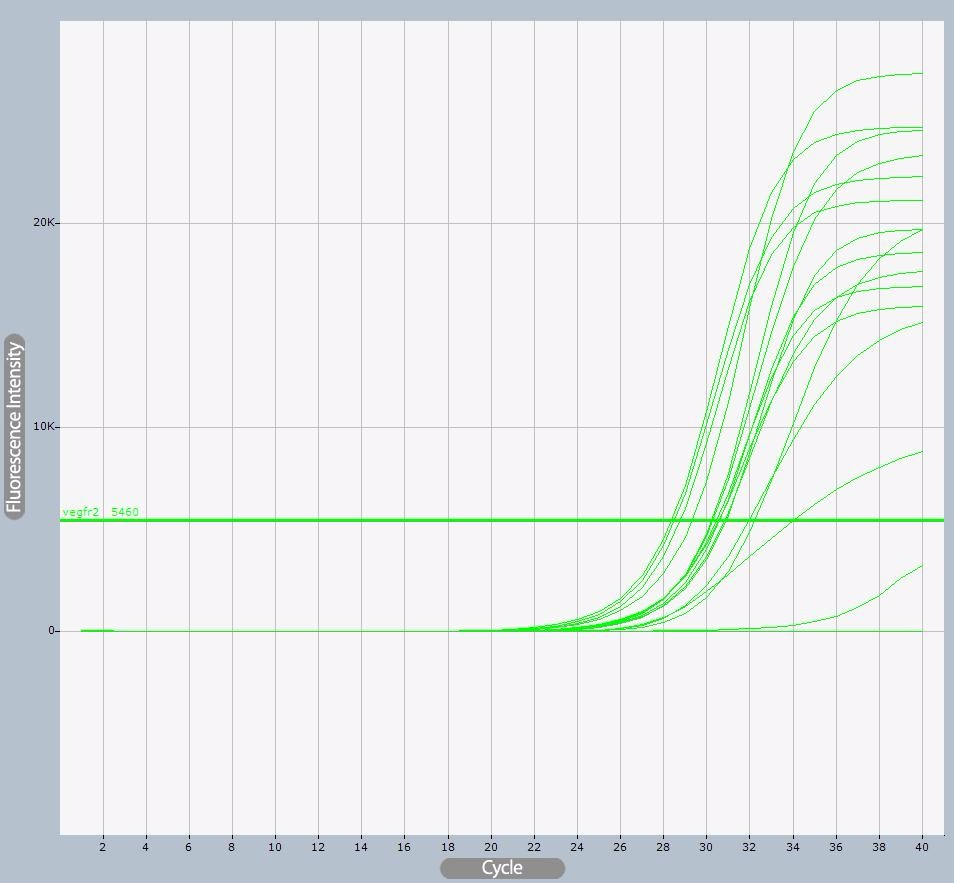


**Supplementary Figure 3** demonstrates the successful amplification of the GAPDH gene in the qPCR assay, with the threshold curve helping to determine the relative quantity of the target gene in the samples. The consistent amplification curves further indicate the reliability of GAPDH as a normalization control in gene expression analyses.

- **ΔCT calculation**

To calculate ΔCT: ΔCT = CT (TSPO) – CT (GAPDH)

A lower ΔCT value indicates higher expression.

This calculation is performed once for a healthy sample and once for a tumor sample. Consequently: ΔΔCT = ΔCT (tumor) – ΔCT (healthy)

A lower ΔΔCT value indicates higher expression.

To determine the fold change:

When comparing gene expression levels across different groups in Real-Time PCR, the best way to represent the change in gene expression in one sample relative to another is to calculate the fold change.

Foldchange = 2^(-ΔΔCT)

A higher fold change value indicates higher expression.

The fold change is calculated once for the healthy sample and once for the tumor sample.


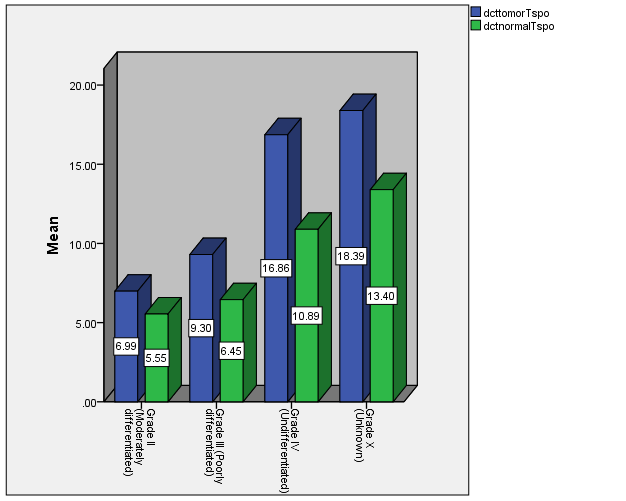


**Supplementary Figure 4.** Chart of TSPO gene expression levels in the tumor and normal groups based on the grade


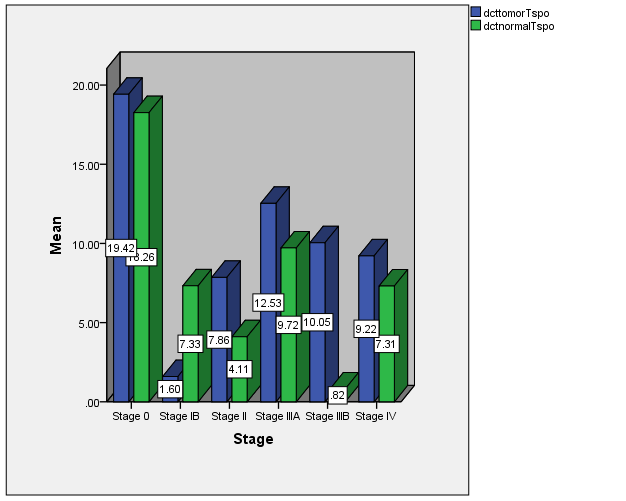


**Supplementary Figure 5.** Chart of TSPO gene expression levels in tumor and normal groups based on the stages.


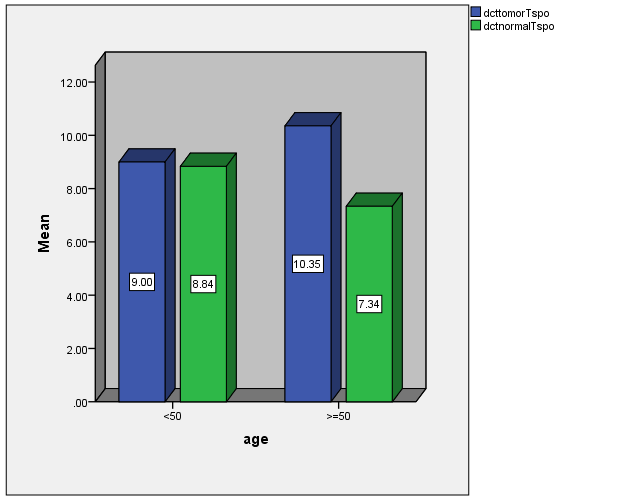


**Supplementary Figure 6.** Chart of TSPO gene expression levels in the tumor and normal groups based on age


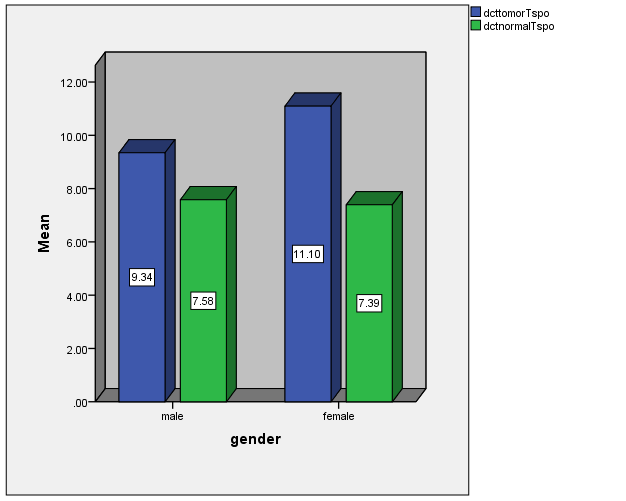


**Supplementary Figure 7.** Chart of TSPO gene expression levels in tumor and normal groups based on gender.
